# *Plasmodium falciparum* Rab1A Localizes to Rhoptries in Schizonts

**DOI:** 10.1371/journal.pone.0158174

**Published:** 2016-06-27

**Authors:** David Morse, Wesley Webster, Ming Kalanon, Gordon Langsley, Geoffrey I. McFadden

**Affiliations:** 1 School of BioSciences, University of Melbourne, Melbourne, VIC, 3010, Australia; 2 Laboratoire de Biologie Cellulaire Comparative des Apicomplexes, Institut Cochin, INSERM U1016, CNRS UMR 8104, Université Paris Descartes, 75014, Paris, France; Ehime University, JAPAN

## Abstract

Over-expression of a GFP-*Pf*Rab1A fusion protein in *Plasmodium falciparum* schizonts produces a punctate pattern of fluorescence typical of rhoptries, secretory organelles involved in host cell invasion. The GFP-positive bodies were purified by a combination of differential and density gradient centrifugation and their protein content determined by MS/MS sequencing. Consistent with the GFP rhoptry-like pattern of transgenic parasites, four of the 19 proteins identified have been previously described to be rhoptry-associated and another four are ER or ER-associated proteins. Confirmation that GFP-*Pf*Rab1A decorates rhoptries was obtained by its co-localization with Rap1 and Ron4 in late phase schizonts. We conclude that *Pf*Rab1A potentially regulates vesicular traffic from the endoplasmic reticulum to the rhoptries in *Apicomplexa* parasites.

## Introduction

The apicomplexan parasite *Plasmodium falciparum* is a causal agent of human malaria. This protist is an important health concern, as according to the 2015 World Health Organization report, it is responsible for roughly 438,000 fatalities yearly worldwide. The parasite has a complex life cycle, involving both insect and human hosts, with mortality mostly a result of parasite replication in the blood [1]. Malaria parasites are an interesting model for protein trafficking, as they contain a number of unusual organelles, including a relict plastid termed an apicoplast [2], thought to be involved in lipid and isoprenoid synthesis and essential for parasite growth [3]. Furthermore, in addition to the mitochondrion, nucleus, ER, Golgi, and food vacuole, there are a number of unique compartments involved in the invasion of host cells. Collectively called the apical complex, a group of three morphologically distinct compartments called the rhoptries [4], micronemes [5], and dense granules [6] are responsible for invasion of blood cells and have a defined choreography of action during the process of host cell invasion [7]. *Plasmodium*, like other members of the *Apicomplexa*, thus contains a number of atypical compartments to which distinct proteins must be specifically targeted.

In general, protein targeting to most of the single membrane-bound compartments in eukaryotes exploits a sophisticated and highly conserved vesicular traffic mechanism. During traffic, vesicles containing cargo are formed at a donor membrane, moved through the cytoplasm to a specific target membrane, and upon fusion with the target membrane release cargo into the new compartment lumen [8, 9]. The determination of the specific destination for a vesicle is critical to the entire process, and is specified by two separate systems of protein-protein interactions. The first involves interactions between SNAREs (Soluble NSF Attachment Receptors) on both the vesicle and target membranes. These interactions not only contribute to the specificity of vesicle docking but also bring the lipid bilayers of the vesicles and the target compartment close enough for fusion to occur [10]. The second protein-protein interaction system involves small monomeric GTPases called Rabs and Rab-binding proteins called Rab effectors or tethers. Tethers can be either large tethering complexes or long coiled-coil proteins, and tethering is thought to precede SNARE binding [11].

The number of Rab isoforms in different cells varies, ranging from 11 (in yeast [12] or *Plasmodium* [13]) to roughly 60 in mammalian cells [14] and *Arabidopsis* [15]. Phylogenetic analysis clusters the different Rabs into ten major groups [16], and at least in some cases, different members of a given group share a conserved function [17, 18]. The functional similarities can extend across species boundaries, as Rab6 isoforms are involved in Golgi targeting in yeast [12] and mammals [19], while Rab5 is endosomal in both yeast [12] and mammals [20]. Rabs are characteristically found associated with the cytoplasmic surface of a particular membrane compartment in the cell, although they can also exist as a soluble protein in the cytoplasm. The GDP-bound form of Rab is soluble in the cytoplasm as a complex with guanine nucleotide dissociation inhibitors (GDI). Rabs become associated with membranes when a GDI displacement factor exposes a prenyl group covalently linked to the C-terminal end of the Rab that then inserts into the membrane. Once freed from the GDI, a guanine nucleotide exchange factor (GEF) on the membrane activates the Rab by exchanging GDP for GTP. Activated Rabs interact with a range of partners, some of which correspond to components of tethering complexes. In addition to vesicle docking, Rabs are also involved in vesicle formation and movement. Interestingly, while *Plasmodium* and yeast both express only 11 Rabs, there are more potential destinations for protein trafficking in *Plasmodium*.

*Plasmodium* has two Rab1 proteins, *Pf*Rab1A and *Pf*Rab1B. *Pf*Rab1B is more closely related to the typical Rab1 found in other organisms than is *Pf*Rab1A. Interestingly, in detailed phylogenetic reconstructions, *Pf*Rab1A appears to be a Rab1 paralog unique to chromalveolates, a phylogenetic group containing the Apicomplexa among others [21]. The function of *Pf*Rab1A has not been extensively studied [13], but in the related apicomplexan *Toxoplasma gondii*, N-terminal myc-tagged *Tg*Rab1A has a punctate appearance and a partial co-localization with markers for an micronemal/endosomal-like compartment, thought to be an intermediate between the Golgi and the apical secretory organelles [22]. To assess the possible role of *Pf*Rab1A, we have examined the distribution of a GFP-*Pf*Rab1A fusion protein in red blood cell stages of *P*. *falciparum*. Similar to what was observed with *Tg*Rab1A, we find that GFP-*Pf*Rab1A has a punctate expression pattern and modest co-localization with micronemal markers. However, we find extensive co-localization of GFP-*Pf*Rab1A with the rhoptry neck marker Ron4 in late phase schizonts. Co-localization is also observed with the rhoptry bulb marker RAP1 in schizonts, suggesting temporal and spatial control over *Pf*Rab1A localization. The punctate distribution of GFP-*Pf*Rab1A and its colocalization with RAP1 in schizonts is also similar to that of the GFP-labeled adaptor protein Mu1 (Pfμ-GFP) [23].

## Methods

### *Pfrab* Cloning

A *Pf*Rab1A clone lacking the N-terminal methionine was generated from *P*. *falciparum* cDNA using a 5' end oligo containing an attB2r site 5'- GGGGACAGCTTTCTTGTACAAAGTGGCTACTGAGAATAGATCAAGAGA-3' and a 3' end oligo containing an attB3 site 5'- GGGGACAACTTTGTATAATAAAGTTGCTTAACAGGAACAAAAGGATTG-3' (*Pfrab1a* sequences underlined). The PCR fragments were cloned into pDONR2r/3 using a gateway BP reaction and their identity confirmed by sequence. This clone was used to generate a CRT5'p-GFP-*Pf*Rab1A fusion using existing promoter and GFP gateway clones. To generate a *Pf*Rab1A-3xHA fusion, *Pfrab1a* clones lacking the terminator codon were amplified using a 5' end oligo containing an attB1 site 5'-GGGGACAAGTTTGTACAAAAAAGCAGGCTTAAAGAAAAAATGACTGAGAATAG-3' and a 3' end oligo containing an attB2 site 5'-GGGGACCACTTTGTACAAGAAAGCTGGGTAACAGGAACAAAAGGATTGAGGA-3'.

PCR fragments were cloned into pDONR2r/3 using a gateway BP reaction and their identity confirmed by sequence. The CRT5'p-GFP-*Pf*Rab1A and the CRT5'p-*Pf*Rab1A-3xHA were produced by Gateway LR reactions and the final clones transfected into the D10 strain *P*. *falciparum*.

A *Pfrab1a* S24N dominant negative (DN) mutant [24] was generated using mutant primers 5'-GGTGTTGGTAAAAATTGTATTTTATTAC and 5'-GTAATAAAATACAATTTTTACCAACACC-3' that, together with the same oligonucleotides used to produce the GFP-PfRab1A fusion, resulted in amplification of a 100 bp mutated 5' end and a 500 bp mutated 3' end. These two fragments were gel purified, mixed and amplified using only the oligonucleotides originally used to produce the GFP-*Pf*Rab1A fusion. The mutant *Pf*Rab1A was cloned into an entry vector with a BP reaction, sequenced, and used to construct an Hsp86p-FKBP-GFP-*Pf*Rab1A-DN using an LR reaction with an FKBP-GFP fusion in a gateway vector. FKBP allows protein levels to be controlled by varying concentrations of the ligand Shield-1 [25].

GFP-*Pf*Rab18 fusions were produced using similar methodology, except that primers for creating the GFP-*Pf*Rab fusion were 5'-GGGGACAGCTTTCTTGTACAAAGTGGCTAAAAATAAAAATAAGTATGATTATTTAC-3' and 5'-GGGGACAACTTTGTATAATAAAGTTGCTTAACAAGCGCAATTGGATCG-3'. These PCR products were used to create an entry vector using a BP reaction and the constructs CRT5'p-GFP-*Pf*Rab1A and Hsp86p-GFP-PfRab1A using an LR reaction. Internal primers for the creation of a *Pf*Rab18 S24N dominant negative mutant were 5'-GTAGGAAAGAATAGTATATTA-3' and 5'-TAATATACTATTCTTTCCTAC-3'. All constructs were sequenced before use. Despite repeated attempts, no transformants were recovered with either of the two FKBP-tagged DN *Pf*Rabs.

### Immunofluorescence Assays (IFA)

IFA was carried out using parasite-infected red blood cells fixed with 4% paraformaldehyde and 0.075% glutaraldehyde [26]. Antibody against the apicoplast marker acyl carrier protein (ACP) was described previously [27], while antibodies against the ER marker BiP and the cis-Golgi marker ERD2 (ER-retention defective complementation group 2) were obtained from the Malaria Research and Reference Reagent Resource Center (MR4). Golgi re-assembly stacking protein (GRASP) antibody was obtained from Tim Gilberger (Hamburg, Germany), and ring-associated erythrocyte surface antigen (RESA) antibody was obtained from Robin Anders (La Trobe, Australia). Antibodies against the micronemal proteins erythrocyte-binding protein 175 (EBA175) and apical membrane antigen 1 (AMA1), as well as against the rhoptry associated protein 1 (Rap1) and a rhoptry neck protein (Ron4) were obtained from Alan Cowman (WEHI, Melbourne Australia). Primary antibodies were visualized using the appropriate Alexa Fluor conjugated secondary antibodies (Molecular probes, Eugene Oregon) and a Leica confocal SP2 microscope.

### Purification of PfRab1A Containing Bodies

Sorbitol synchronized parasites expressing GFP-*Pf*Rab1A were harvested at roughly 5% parasitemia by saponin lysis (0.15% saponin, 0.1% BSA in PBS, 10 minutes on ice), and were washed three times in ice cold PBS. The final pellet was resuspended in 1 mL cold TESP (20 mM Tris pH 7.4, 5 mM EDTA, 0.25 M sucrose and complete protease inhibitor cocktail (Roche)). Cells were lysed by sonication (10 sec burst at 20% power) using a Braun sonicator, and the lysate centrifuged in an Eppendorf microcentrifuge at 4°C three times at 5,000 g for 5 min, then once at 13,000 g for 30 min. The final pellet was resuspended in 100 μL TESP and layered on top of a Percoll step gradient containing 0.25 mL 45% Percoll, 0.5 mL 22.5% Percoll and 0.25 mL 5% Percoll in TESP. The samples were centrifuged at 13,000 g for 30 min at 4°C and fractions taken from the top. All samples were examined microscopically for the presence of GFP fluorescence, which was found between blue (1.037 g/mL) and yellow-green (1.054 g/mL) density marker beads. This sample was diluted ten times with TES, and pelleted by centrifugation at 4°C in an Eppendorf at 13,000 g for 30 min. Cell pellets were digested with trypsin for proteomic analysis.

### Parasite Invasion Estimation

Synchronized cultures of *P*. *falciparum* expressing a given GFP-fusion were diluted to 1% parasitemia and the percentage of red blood cells with rings counted microscopically starting 24 h later. Ten microscope fields were counted for each time point.

## Results and Discussion

**Fig 1:**
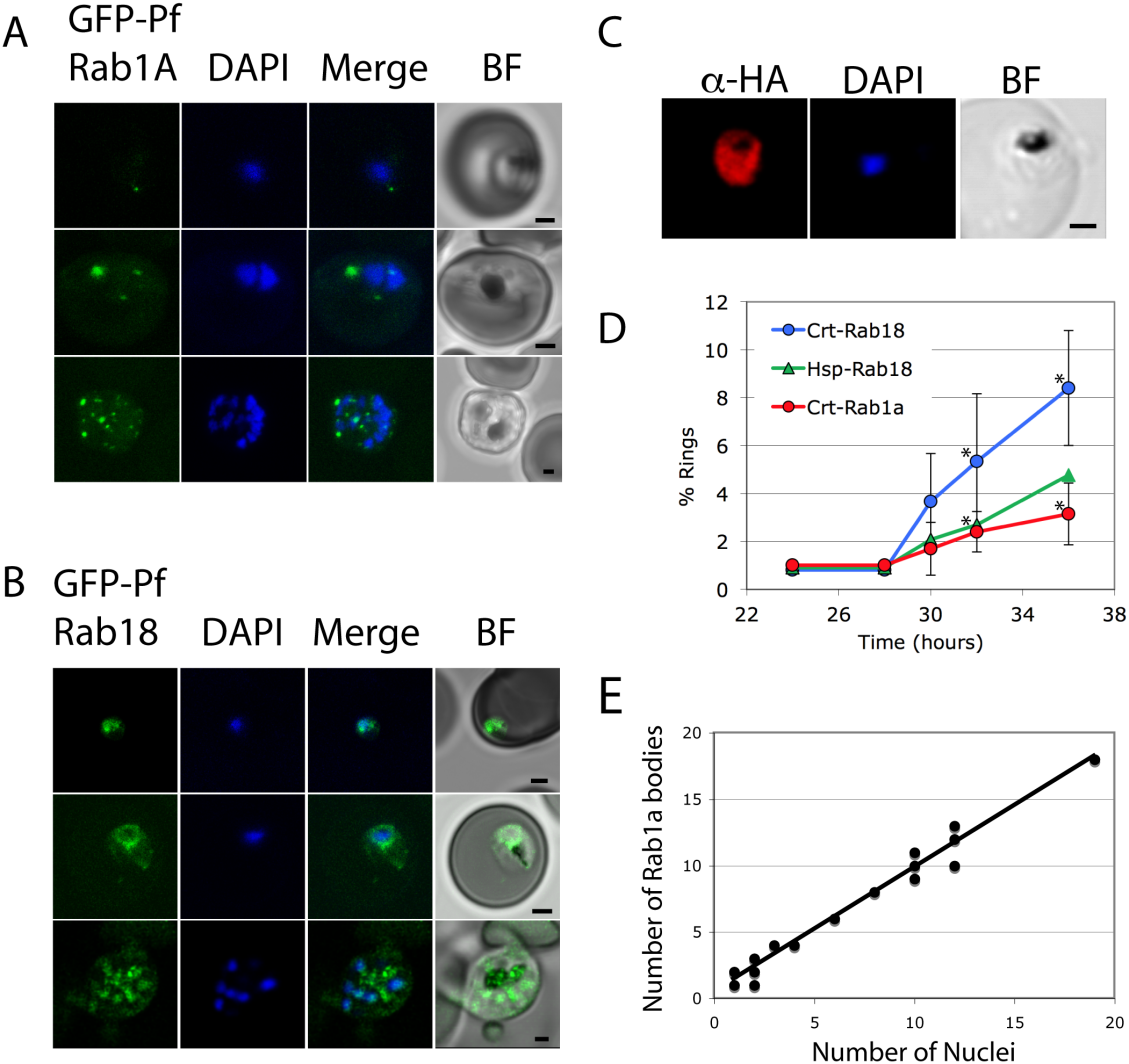
GFP-*Pf*Rab1A fluorescence is found as discrete loci in living cells. A. GFP fluorescence is observed as discrete loci termed *Pf*Rab1A bodies in trophozoite and schizont stage parasites expressing GFP-*Pf*Rab1A. B. GFP-*Pf*Rab18 fluorescence is diffuse and closely associated with the nuclei. C. C-terminal HA-tagged *Pf*Rab1A is distributed equally throughout the parasite cytoplasm. D. Infection by parasites expressing GFP-*Pf*Rab1A is less efficient than parasites expressing GFP-*Pf*Rab18 from the same promoter. Asterisks show significant differences for each time (p ≤ 0.01) using Student’s unpaired two-tailed t-test. E. While not associated with the nucleus, the number of GFP-*Pf*Rab1A loci is proportional to the number of nuclei in the cell.

To assess possible roles for *Pf*Rab1A, a GFP-*Pf*Rab1A fusion was expressed in *P*. *falciparum*. The GFP-fluorescence of transgenic parasites appears localized to discrete loci (Fig 1A), which we term *Pf*Rab1A bodies. This distribution appears specific, as it differs from that produced by GFP-*Pf*Rab18 (Fig 1B), which is found to be more diffuse and close to the nucleus. The specificity of GFP-*Pf*Rab1A also depends on its correct geranylgeranylation, as when the C-terminal prenylation motif is ablated by addition of a hemaglutinin-tag the *Pf*Rab1A-HA fluorescence becomes defuse (Fig 1C). Interestingly, both Crt5’p-GFP-*Pf*Rab1A and Hsp86p-*Pf*Rab18 transgenic parasites display dampened ability to invade new red blood cells when compared to the CRT5’p-GFP-*Pf*Rab18 (Fig 1D), indicating that upon over-expression of GFP-*Pf*Rab1A, secretory organelle function may be impaired. The number of *Pf*Rab1A bodies increases proportionately with the number of nuclei (Fig 1E) with a *Pf*Rab1A body to nucleus ratio of 1.1 ± 0.4. A similar punctate staining pattern and an increasing number of fluorescent bodies with number of nuclei has also been observed in *P*. *falciparum* expressing Pfμ-GFP, an adaptor protein involved in rhoptry protein trafficking from the Golgi [23]. The punctate pattern in schizonts is also seen using GFP fused to the rhoptry marker RAP1 [28].

An issue that must be addressed when using an over-expressed GFP-*Pf*Rab1A fusion protein is that Rab localization may be affected by the degree of expression. In some cases, Rab overexpression can alter the number or size of a target compartment [29, 30], or provoke a mistargeting of cargo proteins [31, 32]. We attempted to address this experimentally by preparing a titratable FKBP-GFP-*Pf*Rab1A and by testing if an epitope tag could be added to the C-terminal end of the protein so as to allow the endogenous gene to be tagged by a 3' replacement strategy. Unfortunately, no transformants were detected in culture when the FKBP-GFP-*Pf*Rab1A was selected for, and the C terminal HA-tag blocked the association of the *Pf*Rab1A-3HA with a target membrane as expected (Fig 1C). However, there are two indirect lines of evidence to suggest the localization of the GFP-*Pf*Rab1A fusion observed here does indeed reflect that of the endogenous protein. First, we note a similar distribution pattern of our GFP-*Pf*Rab1A and the FKBP-*Tg*Rab1A in *Toxoplasma gondii* [22]. In the latter case, the FKBP moiety allowed the FKBP-*Tg*Rab1A fusion to be titrated down to the lowest levels compatible with detection in *Toxoplasma*. Second, Rab mistargeting can often impair a cell’s ability to function properly [31], yet the cells expressing either GFP-*Pf*Rab1A (Fig 1D) or FKBP-*Tg*Rab1A [22] proliferate, albeit slightly less well than wild type cells. Unfortunately, our DN FKBP-*Pf*Rab1A lines did not proliferate at all.

The function of *Pf*Rab1A is still unclear. In plants and animals, *Pf*Rab1A orthologs typically have an ER/Golgi/endosome location and the generally accepted view is that *Pf*Rab1A functions in ER to Golgi traffic [33, 34]. However, several studies suggest that the role of *Pf*Rab1A may be more complex. It has been found to be associated with transcytotic vesicles [35], and has also been implicated in a novel pathway linking ER with the cell periphery [36]. In *Apicomplexa* the role of *Pf*Rab1A is further complicated by molecular phylogenetic reconstructions showing it is distinct from Rab1A of higher plants or animals (S1 Fig) [21].

**Fig 2:**
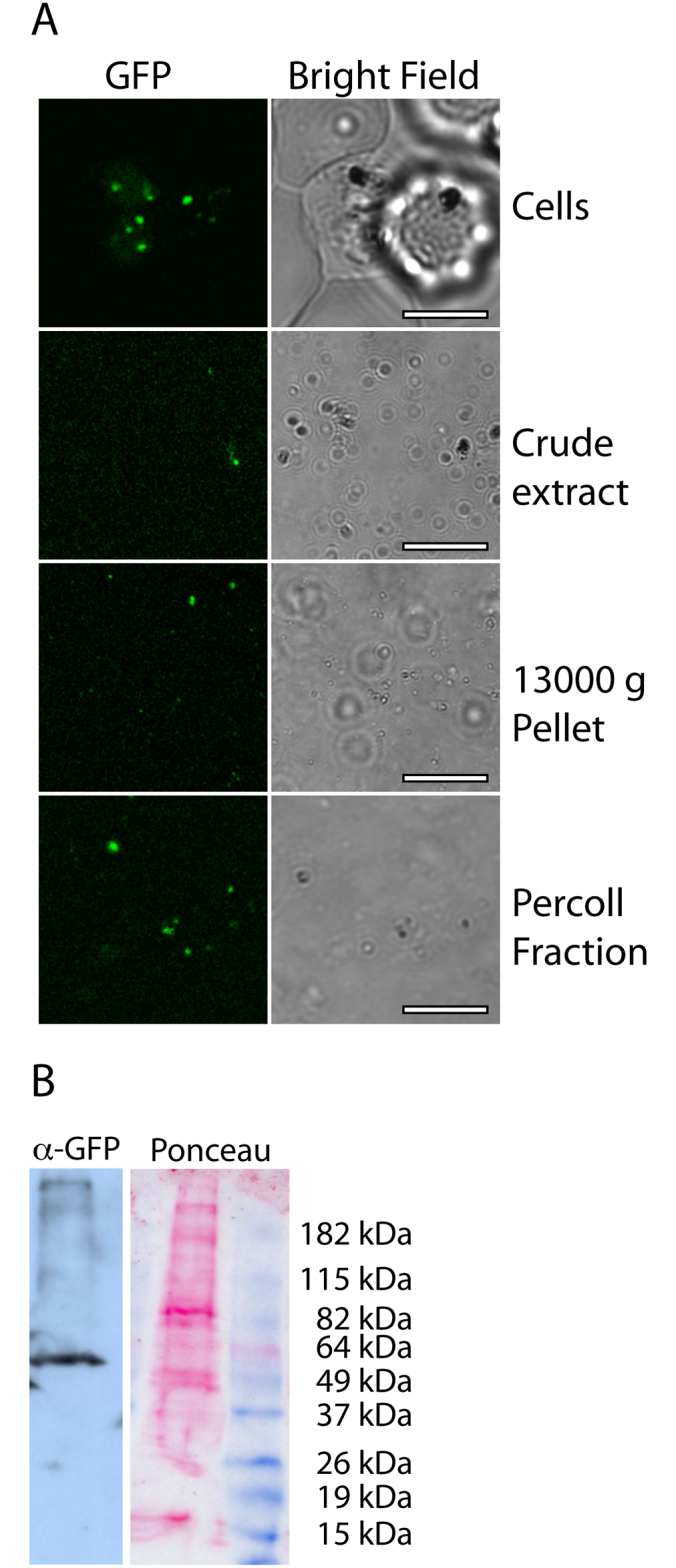
Purification of GFP-*Pf*Rab1A bodies. Samples taken from different stages of purification, starting with whole cells (top), and including a crude cell extract, a high speed (13,000 g) centrifugation pellet, and a fraction from a Percoll density gradient spanning 1.037 to 1.054 g/mL subsequently pelleted by centrifugation at 13,000 g. Scale bars are 3 μm. (B) Western blot using an antibody against GFP on the purified Percoll fraction shows a signal at a size (~55 kDa) consistent with GFP-*Pf*Rab1A even though the protein levels of the fusion are insufficient to visualize by Ponceau staining.

As a guide to determining the proteins that might be associated with *Pf*Rab1A bodies, we sequenced proteins associated with a partially purified GFP-positive body fraction from sorbitol-synchronized late trophozoite/early schizont stages. The purification used a combination of differential centrifugation and Percoll density gradient centrifugation. The presence of GFP-*Pf*Rab1A was followed throughout fractionation using fluorescence microscopy (Fig 2A) and the presence of GFP-*Pf*Rab1A in the purified fraction was confirmed by Western analysis with anti-GFP (Fig 2B). Nineteen proteins were identified by LC-MS/MS analyses in the purified fraction (≥2 peptides from each candidate, Table 1; S1 Table). This analysis identified four rhoptry proteins together with Sortilin, a cargo receptor involved in vesicular trafficking. In *T*. *gondii*, Sortilin-like receptor TgSORTLR is essential for transport of proteins to both micronemes and rhoptries [37]. TgSORTLR is a membrane protein localized to the Golgi/endosomes whose cytoplasmic C-terminal end binds a variety of vesicular coat proteins and whose N-terminal domain binds a variety of micronemal and rhoptry proteins.

**Table 1:** Proteomic analysis of purified PfRab1A bodies.

Identification	Gene	MW (kD)	Peptides	Features^1^	DRM^2^
Multidrug resistance protein	PF3D7_0523000	162.2	2	PM	+
Vacuolar proton-translocating ATPase subunit A, putative	PF3D7_0806800	123	2	PM	+
Merozoite surface protein 1 precursor	PF3D7_0930300	193.7	6	PM	+
Hypothetical protein	PF3D7_1462300	161	2	SP, 3TMD	
MSP7-like	PF3D7_1334500	75.5	2	SP, 1TMD	
Sortilin, putative	PF3D7_1451800	102.2	2	Golgi/Endo	
RhopH3	PF3D7_0905400	104.8	2	Rhop	+
RhopH2	PF3D7_0929400	161	2	Rhop	+
Rap1	PF3D7_1410400	90	6	Rhop	+
Circumsporozoite protein-related antigen	PF3D7_1121600	17.3	3	Rhop	+
Heat shock protein Pfhsp70-2	PF3D7_0917900	72.4	11	ER	+
Endoplasmic reticulum-resident calcium binding protein	PF3D7_1108600	39.4	4	ER	
Endoplasmin homolog precursor, putative (hsp90)	PF3D7_1222300	95	9	ER	
Heat shock protein Pfhsp70-3	PF3D7_1134000	71.6	3	Cyt	+
Elongation factor 1 alpha	PF3D7_1357000	48.9	4	Cyt	+
Hsp60	PF3D7_1015600	62.5	2	Cyt	
VAMP-associated protein A	PF3D7_1439800	27.7	2	Cyt	
Histone H4	PF3D7_1105000	11.4	3		+
Histone H2B	PF3D7_1105100	13.1	2		

^1^ PM, plasma membrane; SP, signal peptide; TMD, transmembrane domain; Rhop, rhoptry; Endo, endosome; ER, endoplasmic reticulum; Cyt, cytoplasm

^2^ Proteins present in a detergent-resistant membrane (GPI-anchored protein) fraction [42].

Among the other proteins associated with *Pf*Rab1A bodies, three are known to be plasma membrane proteins, and the two hypothetical proteins found are likely to be integral membrane proteins given that they both contain a signal peptide and one or more transmembrane domains (Table 1). While none of these are known rhoptry constituents, it is certainly plausible that their mechanism of trafficking inside the parasite may involve *Pf*Rab1A. In addition, there are three ER proteins found among the purified proteins. The presence of ER proteins is interesting, as the usual view of rhoptry formation involves transport of proteins from a Golgi/endosome compartment. The presence of the VAMP-associated membrane protein A (VAP-A) is also of interest, as this protein binds SNAREs and is thus also likely to be involved in vesicle trafficking [38].

However, it is possible that association of at least some of the proteins found in the *Pf*Rab1A body fraction may be due to non-specific interactions. Two (Histone H4 and the endoplasmic reticulum resident calcium binding protein) have been found to be promiscuous interactants during an extensive two-hybrid screen to characterize the *Plasmodium* interactome [39]. It also seems likely that the different heat shock proteins may be non-specifically associated with the *Pf*Rab1A body, given the large number of interactants that have been reported for these proteins [40]. It is also unlikely that EF1 alpha will be a specific interactant with the compartment, as it is emerging as an abundant cytoplasmic protein capable of multiple interactions [41]. If these potentially non-specific interactants were to be excluded from the analysis, the proportion of the *Pf*Rab1A body proteome that is rhoptry-associated will evidently increase.

Interestingly, 11 of the 19 proteins identified in *Pf*Rab1A bodies were also found in a detergent-resistant membrane (DRM) fraction obtained from merozoites [42]. In plants and fungi, DRM fractions are thought to represent specialized regions of the plasma membrane important for cell-cell interactions. This would certainly be consistent with the presence of GPI-anchored, transmembrane and rhoptry proteins found in the *Plasmodium* DRM fraction. For example, the GPI-anchored protein RAMA in the DRM fraction is known to be targeted to the rhoptry and may be responsible for binding and trafficking of other rhoptry proteins such as Rap1 [28].

**Fig 3:**
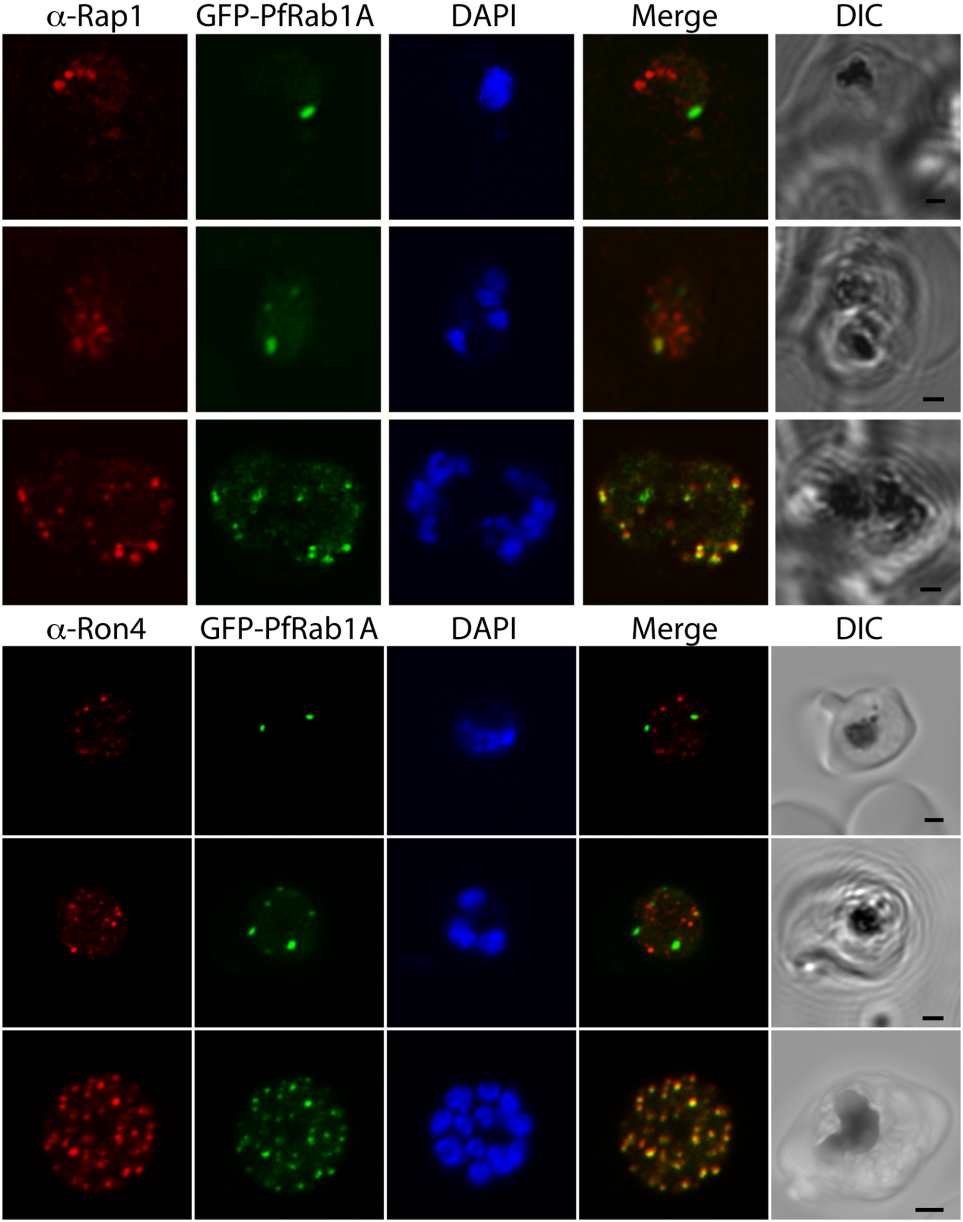
GFP-*Pf*Rab1A fluorescence in late schizonts is associated with rhoptry markers. Rhoptry markers Rap1 and Ron4 are found in trophozoites and schizonts as discrete foci that colocalize with GFP-*Pf*Rab1A fluorescence in late schizonts, but not in earlier phases of parasite development.

Given the number of rhoptry proteins detected in the purified GFP-*Pf*Rab1A fraction, we elected to confirm the association of GFP-*Pf*Rab1A with rhoptries using immunocytochemistry. We observe extensive colocalization of GFP fluorescence with Rap1 and Ron4 in late stages of schizonts (Fig 3). A slight difference in overlap was observed with the rhoptry bulb marker Rap1 compared to the rhoptry neck marker Ron4, consistent with the presence of two distinct domains within the organelle [43]. There is no co-localization between GFP-*Pf*Rab1A and ER markers Bip, ERD2, or GRASP, nor is there colocalization after staining with Bodipy-BFA, a fluorescent version of Brefeldin A (S2 Fig). However, modest co-localization was observed with the microneme markers AMA1 and EBA175. This staining pattern is thus consistent with the observed colocalization pattern of FKBP-*Tg*Rab1A [22].

The biogenesis of the rhoptry occurs late during intraerythrocytic development and coincides with late-stage expression of the rhoptry proteins RhopH1,2,3 [44]. In agreement with its nature as a compartment likely related to a secretory lysosome, as well as with a general use of clathrin-coated vesicles for targeting lysosomes in animal cells, AP-1 adaptin has been implicated in transport to the *Toxoplasma* rhoptry [45]. Furthermore, in cells mutated in the dynamin related protein DrpB expression, rhoptries do not form and rhoptry proteins are mistargeted to the constitutive secretory pathway [46]. However, an alternative pathway for protein targeting, involving rhoptry associated membrane antigen (RAMA) has also been reported [28]. Rhoptries contain at least two distinct regions, the neck and the bulb, which can be distinguished both morphologically and by the presence of different protein markers [4]. During development of the rhoptry in the schizonts, the bulb appears first and results from fusion of vesicles originating from the Golgi [47]. At later stages the rhoptry neck forms, apparently due to vesicular traffic to the neck region directly. If the neck protein Ron4 is targeted to the neck subsequent to establishment of the neck structure, this could explain the colocalization of GFP-*Pf*Rab1A and Ron4 seen in schizonts, but not in earlier stage parasites.

We present here the colocalization of GFP-*Pf*Rab1A with rhoptry markers using immunofluorescence assays. The possibility that a *Pf*Rab may be involved in targeting proteins to rhoptries, in addition to clathrin coated vesicle targeting, is consistent with recent findings that proteins may be targeted to the secretory organelles by several different routes [22, 28] and certainly warrants further study. Such studies could include more exhaustive colocalization studies using TEM or super-resolution microscopy.

## Supporting Information

S1 Fig The phylogenetic position of *Pf*Rab1A differs from the *Pf*Rab1B. Phlylogenetic analysis of the 11 *Plasmodium falciparum* Rab proteins (black) with selected examples of major Rab family members from mammals (mouse; red) and plants (*Arabidopsis*; green) as well as the 11 budding yeast Rabs (blue). Note, inclusion of *Pf*Rab1A in group I-D is not strongly supported by bootstrap analysis.
(TIF)

S2 Fig GFP-*Pf*Rab1A fluorescence does not co-localize with ER/Golgi, dense granules or microneme markers. GFP-*Pf*Rab1A fluorescence is distinct from the localization of markers for the apicoplast (ACP), the ER (Bip), the Golgi (ERD2 and GRASP), as well as from staining of the ER/Golgi with Bodipy BFA. GFP-*Pf*Rab1A fluorescence is also distinct from the localization of markers for dense granules (RESA) or micronemes (AMA1 and EBA175).
(PDF)

S1 Table Peptide sequences recovered from the GFP-*Pf*Rab1A enriched fraction. (PDF)

## References

[1] Cowman AF, Crabb BS. Invasion of red blood cells by malaria parasites. Cell. 2006;124(4):755–66. 10.1016/j.cell.2006.02.006 . 16497586

[2] Waller RF, McFadden GI. The apicoplast: a review of the derived plastid of apicomplexan parasites. Current issues in molecular biology. 2005;7(1):57–79. . 15580780

[3] Ralph SA, Van Dooren GG, Waller RF, Crawford MJ, Fraunholz MJ, Foth BJ, et al Tropical infectious diseases: Metabolic maps and functions of the Plasmodium falciparum apicoplast. Nature reviews Microbiology. 2004;2(3):203–16. . 15083156 10.1038/nrmicro843

[4] Kats LM, Black CG, Proellocks NI, Coppel RL. Plasmodium rhoptries: how things went pear-shaped. Trends in parasitology. 2006;22(6):269–76. 10.1016/j.pt.2006.04.001 . 16635585

[5] Soldati D, Dubremetz JF, Lebrun M. Microneme proteins: structural and functional requirements to promote adhesion and invasion by the apicomplexan parasite Toxoplasma gondii. Int J Parasitol. 2001;31(12):1293–302. . 11566297 10.1016/s0020-7519(01)00257-0

[6] Mercier C, Adjogble KD, Daubener W, Delauw MF. Dense granules: are they key organelles to help understand the parasitophorous vacuole of all apicomplexa parasites? Int J Parasitol. 2005;35(8):829–49. 10.1016/j.ijpara.2005.03.011 . 15978597

[7] Counihan NA, Kalanon M, Coppel RL, de Koning-Ward TF. Plasmodium rhoptry proteins: why order is important. Trends in parasitology. 2013;29(5):228–36. 10.1016/j.pt.2013.03.003 . 23570755

[8] Bonifacino JS, Glick BS. The mechanisms of vesicle budding and fusion. Cell. 2004;116(2):153–66. . 14744428 10.1016/s0092-8674(03)01079-1

[9] van Vliet C, Thomas EC, Merino-Trigo A, Teasdale RD, Gleeson PA. Intracellular sorting and transport of proteins. Prog Biophys Mol Biol. 2003;83(1):1–45. . 12757749 10.1016/s0079-6107(03)00019-1

[10] Sudhof TC, Rothman JE. Membrane fusion: grappling with SNARE and SM proteins. Science. 2009;323(5913):474–7. 10.1126/science.1161748 19164740 PMC3736821

[11] Cai H, Reinisch K, Ferro-Novick S. Coats, tethers, Rabs, and SNAREs work together to mediate the intracellular destination of a transport vesicle. Dev Cell. 2007;12(5):671–82. . 17488620 10.1016/j.devcel.2007.04.005

[12] Buvelot Frei S, Rahl PB, Nussbaum M, Briggs BJ, Calero M, Janeczko S, et al Bioinformatic and comparative localization of Rab proteins reveals functional insights into the uncharacterized GTPases Ypt10p and Ypt11p. Mol Cell Biol. 2006;26(19):7299–317. . 16980630 10.1128/MCB.02405-05 PMC1592887

[13] Quevillon E, Spielmann T, Brahimi K, Chattopadhyay D, Yeramian E, Langsley G. The Plasmodium falciparum family of Rab GTPases. Gene. 2003;306:13–25. . 12657463 10.1016/s0378-1119(03)00381-0

[14] Stenmark H, Olkkonen VM. The Rab GTPase family. Genome biology. 2001;2(5):REVIEWS3007 11387043 10.1186/gb-2001-2-5-reviews3007 PMC138937

[15] Rutherford S, Moore I. The Arabidopsis Rab GTPase family: another enigma variation. Current opinion in plant biology. 2002;5(6):518–28. . 12393015 10.1016/s1369-5266(02)00307-2

[16] Collins RN. Application of phylogenetic algorithms to assess Rab functional relationships. Methods Enzymol. 2005;403:19–28. . 16473574 10.1016/S0076-6879(05)03003-X

[17] Segev N, Mulholland J, Botstein D. The yeast GTP-binding YPT1 protein and a mammalian counterpart are associated with the secretion machinery. Cell. 1988;52(6):915–24. . 3127057 10.1016/0092-8674(88)90433-3

[18] Singer-Kruger B, Stenmark H, Zerial M. Yeast Ypt51p and mammalian Rab5: counterparts with similar function in the early endocytic pathway. J Cell Sci. 1995;108 (Pt 11):3509–21. . 8586662 10.1242/jcs.108.11.3509

[19] Mallard F, Tang BL, Galli T, Tenza D, Saint-Pol A, Yue X, et al Early/recycling endosomes-to-TGN transport involves two SNARE complexes and a Rab6 isoform. J Cell Biol. 2002;156(4):653–64. . 11839770 10.1083/jcb.200110081 PMC2174079

[20] Bucci C, Parton RG, Mather IH, Stunnenberg H, Simons K, Hoflack B, et al The small GTPase rab5 functions as a regulatory factor in the early endocytic pathway. Cell. 1992;70(5):715–28. . 1516130 10.1016/0092-8674(92)90306-w

[21] Elias M, Patron NJ, Keeling PJ. The RAB Family GTPase Rab1A from Plasmodium falciparum Defines a Unique Paralog Shared by Chromalveolates and Rhizaria. Journal of Eukaryotic Microbiology. 2009;56(4):348–56. 10.1111/j.1550-7408.2009.00408.x 19602080

[22] Kremer K, Kamin D, Rittweger E, Wilkes J, Flammer H, Mahler S, et al An overexpression screen of Toxoplasma gondii Rab-GTPases reveals distinct transport routes to the micronemes. PLoS pathogens. 2013;9(3):e1003213 10.1371/journal.ppat.1003213 23505371 PMC3591302

[23] Kaderi Kibria KM, Rawat K, Klinger CM, Datta G, Panchal M, Singh S, et al A role for adaptor protein complex 1 in protein targeting to rhoptry organelles in Plasmodium falciparum. Biochim Biophys Acta. 2015;1853(3):699–710. 10.1016/j.bbamcr.2014.12.030 . 25573429

[24] Zhang J, Schulze KL, Hiesinger PR, Suyama K, Wang S, Fish M, et al Thirty-one flavors of Drosophila rab proteins. Genetics. 2007;176(2):1307–22. 10.1534/genetics.106.066761 17409086 PMC1894592

[25] Herm-Gotz A, Agop-Nersesian C, Munter S, Grimley JS, Wandless TJ, Frischknecht F, et al Rapid control of protein level in the apicomplexan Toxoplasma gondii. Nat Methods. 2007;4(12):1003–5. 10.1038/nmeth1134 17994029 PMC2601725

[26] Tonkin CJ, van Dooren GG, Spurck TP, Struck NS, Good RT, Handman E, et al Localization of organellar proteins in Plasmodium falciparum using a novel set of transfection vectors and a new immunofluorescence fixation method. Mol Biochem Parasitol. 2004;137(1):13–21. . 15279947 10.1016/j.molbiopara.2004.05.009

[27] Waller RF, Reed MB, Cowman AF, McFadden GI. Protein trafficking to the plastid of Plasmodium falciparum is via the secretory pathway. Embo J. 2000;19(8):1794–802. . 10775264 10.1093/emboj/19.8.1794 PMC302007

[28] Richard D, Kats LM, Langer C, Black CG, Mitri K, Boddey JA, et al Identification of rhoptry trafficking determinants and evidence for a novel sorting mechanism in the malaria parasite Plasmodium falciparum. PLoS pathogens. 2009;5(3):e1000328 10.1371/journal.ppat.1000328 19266084 PMC2648313

[29] Mesa R, Salomon C, Roggero M, Stahl PD, Mayorga LS. Rab22a affects the morphology and function of the endocytic pathway. J Cell Sci. 2001;114(Pt 22):4041–9. . 11739636 10.1242/jcs.114.22.4041

[30] Roberts RL, Barbieri MA, Pryse KM, Chua M, Morisaki JH, Stahl PD. Endosome fusion in living cells overexpressing GFP-rab5. J Cell Sci. 1999;112 (Pt 21):3667–75. . 10523503 10.1242/jcs.112.21.3667

[31] Welter BH, Temesvari LA. Overexpression of a mutant form of EhRabA, a unique Rab GTPase of Entamoeba histolytica, alters endoplasmic reticulum morphology and localization of the Gal/GalNAc adherence lectin. Eukaryot Cell. 2009;8(7):1014–26. 10.1128/EC.00030-09 19377040 PMC2708452

[32] Martinez O, Antony C, Pehau-Arnaudet G, Berger EG, Salamero J, Goud B. GTP-bound forms of rab6 induce the redistribution of Golgi proteins into the endoplasmic reticulum. Proc Natl Acad Sci U S A. 1997;94(5):1828–33. 9050864 10.1073/pnas.94.5.1828 PMC20002

[33] Peter F, Nuoffer C, Pind SN, Balch WE. Guanine nucleotide dissociation inhibitor is essential for Rab1 function in budding from the endoplasmic reticulum and transport through the Golgi stack. J Cell Biol. 1994;126(6):1393–406. . 8089173 10.1083/jcb.126.6.1393 PMC2290957

[34] Saraste J, Lahtinen U, Goud B. Localization of the small GTP-binding protein rab1p to early compartments of the secretory pathway. J Cell Sci. 1995;108 (Pt 4):1541–52. . 7615674 10.1242/jcs.108.4.1541

[35] Jin M, Saucan L, Farquhar MG, Palade GE. Rab1a and multiple other Rab proteins are associated with the transcytotic pathway in rat liver. J Biol Chem. 1996;271(47):30105–13. . 8939959 10.1074/jbc.271.47.30105

[36] Sannerud R, Marie M, Nizak C, Dale HA, Pernet-Gallay K, Perez F, et al Rab1 defines a novel pathway connecting the pre-Golgi intermediate compartment with the cell periphery. Molecular biology of the cell. 2006;17(4):1514–26. . 16421253 10.1091/mbc.E05-08-0792 PMC1415313

[37] Sloves PJ, Delhaye S, Mouveaux T, Werkmeister E, Slomianny C, Hovasse A, et al Toxoplasma sortilin-like receptor regulates protein transport and is essential for apical secretory organelle biogenesis and host infection. Cell host & microbe. 2012;11(5):515–27. 10.1016/j.chom.2012.03.006 . 22607804

[38] Weir ML, Xie H, Klip A, Trimble WS. VAP-A binds promiscuously to both v- and tSNAREs. Biochem Biophys Res Commun. 2001;286(3):616–21. 10.1006/bbrc.2001.5437 . 11511104

[39] LaCount DJ, Vignali M, Chettier R, Phansalkar A, Bell R, Hesselberth JR, et al A protein interaction network of the malaria parasite Plasmodium falciparum. Nature. 2005;438(7064):103–7. 10.1038/nature04104 . 16267556

[40] Gong Y, Kakihara Y, Krogan N, Greenblatt J, Emili A, Zhang Z, et al An atlas of chaperone-protein interactions in Saccharomyces cerevisiae: implications to protein folding pathways in the cell. Molecular systems biology. 2009;5:275 10.1038/msb.2009.26 19536198 PMC2710862

[41] Mateyak MK, Kinzy TG. eEF1A: thinking outside the ribosome. J Biol Chem. 2010;285(28):21209–13. 10.1074/jbc.R110.113795 20444696 PMC2898402

[42] Sanders PR, Gilson PR, Cantin GT, Greenbaum DC, Nebl T, Carucci DJ, et al Distinct protein classes including novel merozoite surface antigens in Raft-like membranes of Plasmodium falciparum. J Biol Chem. 2005;280(48):40169–76. . 16203726 10.1074/jbc.M509631200

[43] Boothroyd JC, Dubremetz JF. Kiss and spit: the dual roles of Toxoplasma rhoptries. Nature reviews Microbiology. 2008;6(1):79–88. 10.1038/nrmicro1800 . 18059289

[44] Jaikaria NS, Rozario C, Ridley RG, Perkins ME. Biogenesis of rhoptry organelles in Plasmodium falciparum. Mol Biochem Parasitol. 1993;57(2):269–79. . 8433718 10.1016/0166-6851(93)90203-a

[45] Ngo HM, Yang M, Paprotka K, Pypaert M, Hoppe H, Joiner KA. AP-1 in Toxoplasma gondii mediates biogenesis of the rhoptry secretory organelle from a post-Golgi compartment. J Biol Chem. 2003;278(7):5343–52. 10.1074/jbc.M208291200 . 12446678

[46] Breinich MS, Ferguson DJ, Foth BJ, van Dooren GG, Lebrun M, Quon DV, et al A dynamin is required for the biogenesis of secretory organelles in Toxoplasma gondii. Curr Biol. 2009;19(4):277–86. 10.1016/j.cub.2009.01.039 19217293 PMC3941470

[47] Bannister LH, Hopkins JM, Fowler RE, Krishna S, Mitchell GH. Ultrastructure of rhoptry development in Plasmodium falciparum erythrocytic schizonts. Parasitology. 2000;121 (Pt 3):273–87. . 11085247 10.1017/s0031182099006320

