# Supplementary figures and images for: Plasmodium falciparum Rab1A Localizes to Rhoptries in Schizonts
Source: PLoS One. 2016 Jun 27;11(6):e0158174. doi: 10.1371/journal.pone.0158174 (PMC4922565; doi:10.1371/journal.pone.0158174)

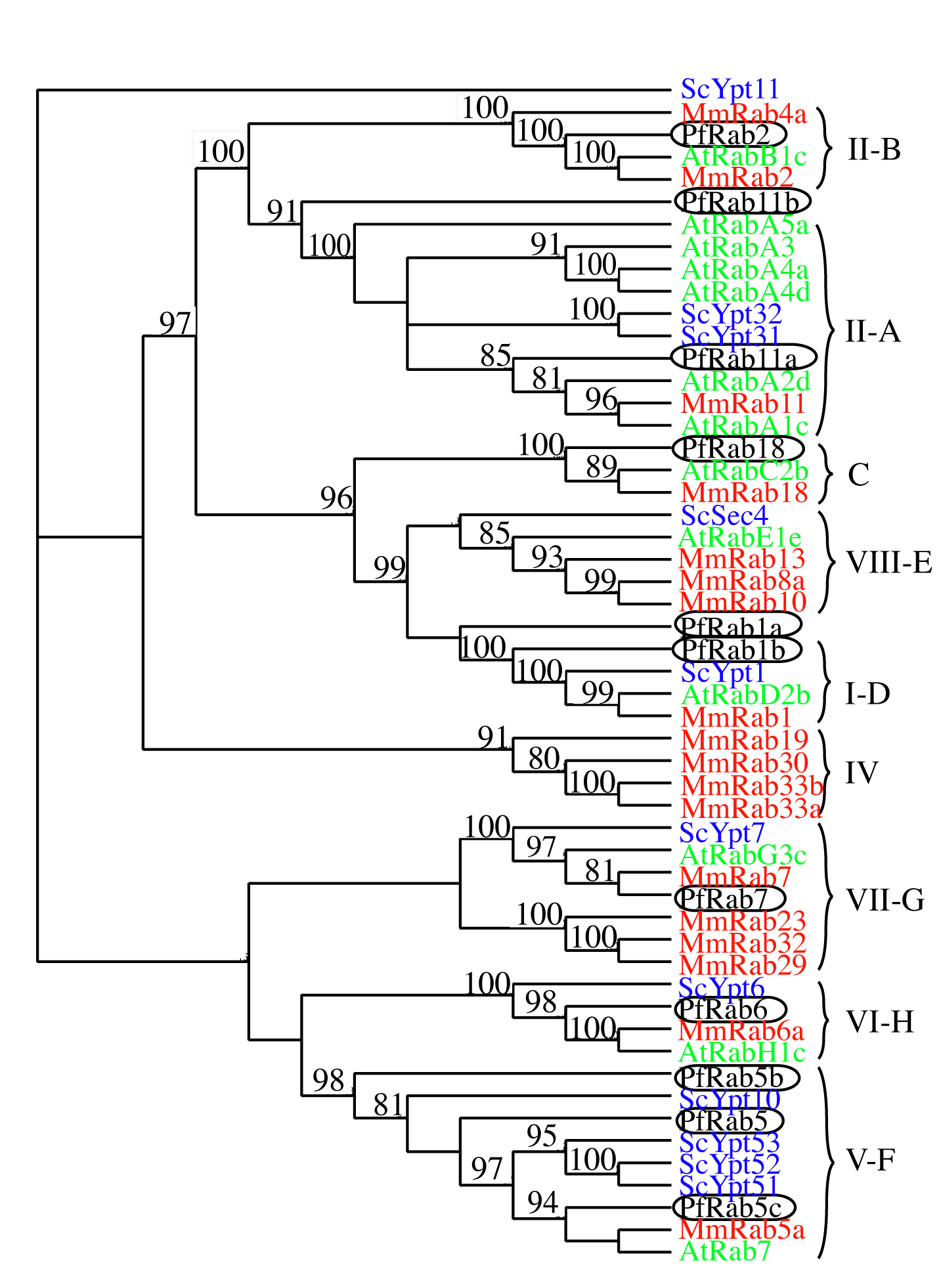

Supplement: S1 Fig — Phlylogenetic analysis of the 11 Plasmodium falciparum Rab proteins (black) with selected examples of major Rab family members from mammals (mouse; red) and plants (Arabidopsis; green) as well as the 11 budding yeast Rabs (blue). Note, inclusion of PfRab1A in group I-D is not strongly supported by bootstrap analysis. (TIF) [file pone.0158174.s001.tif]

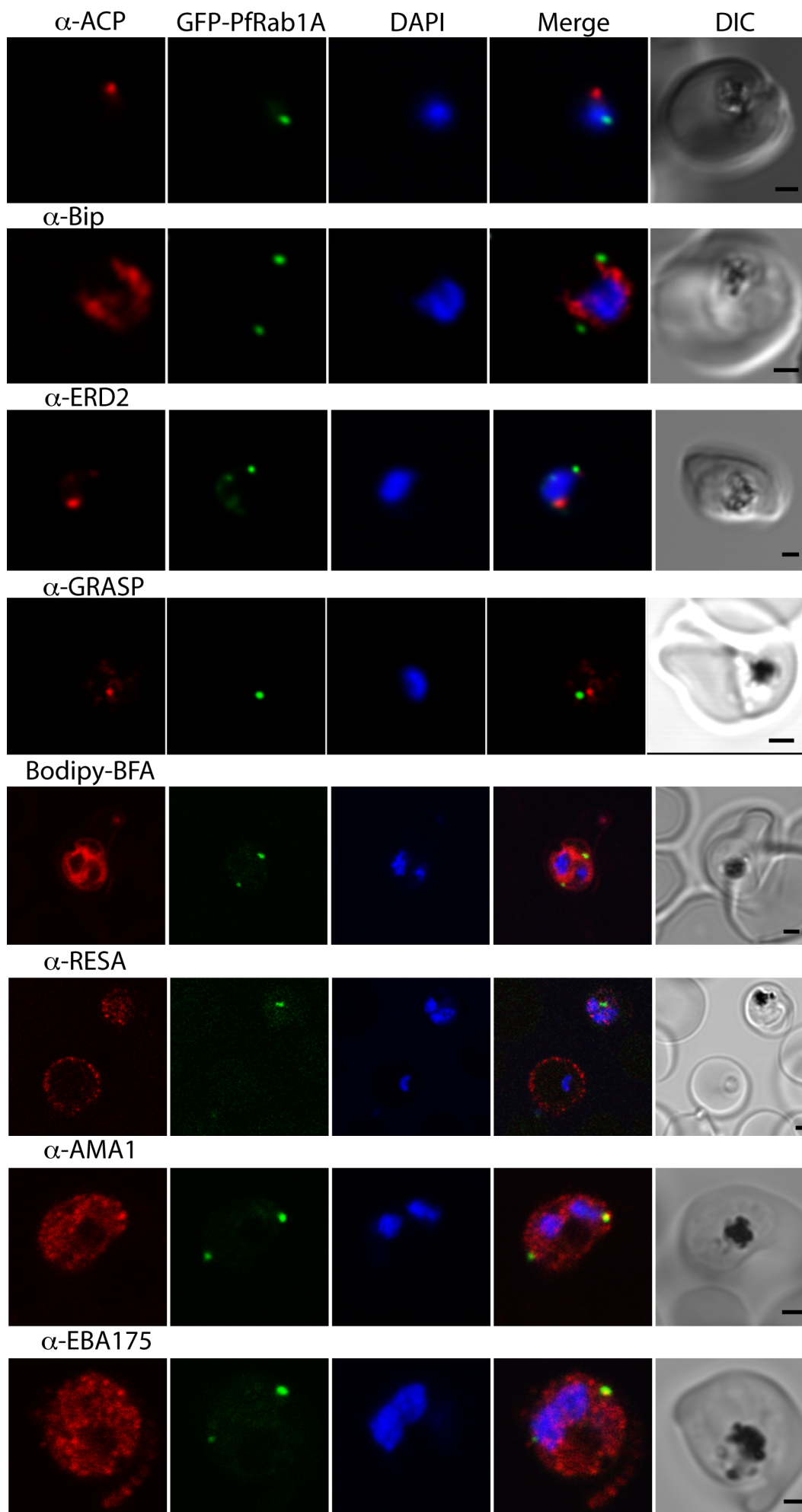

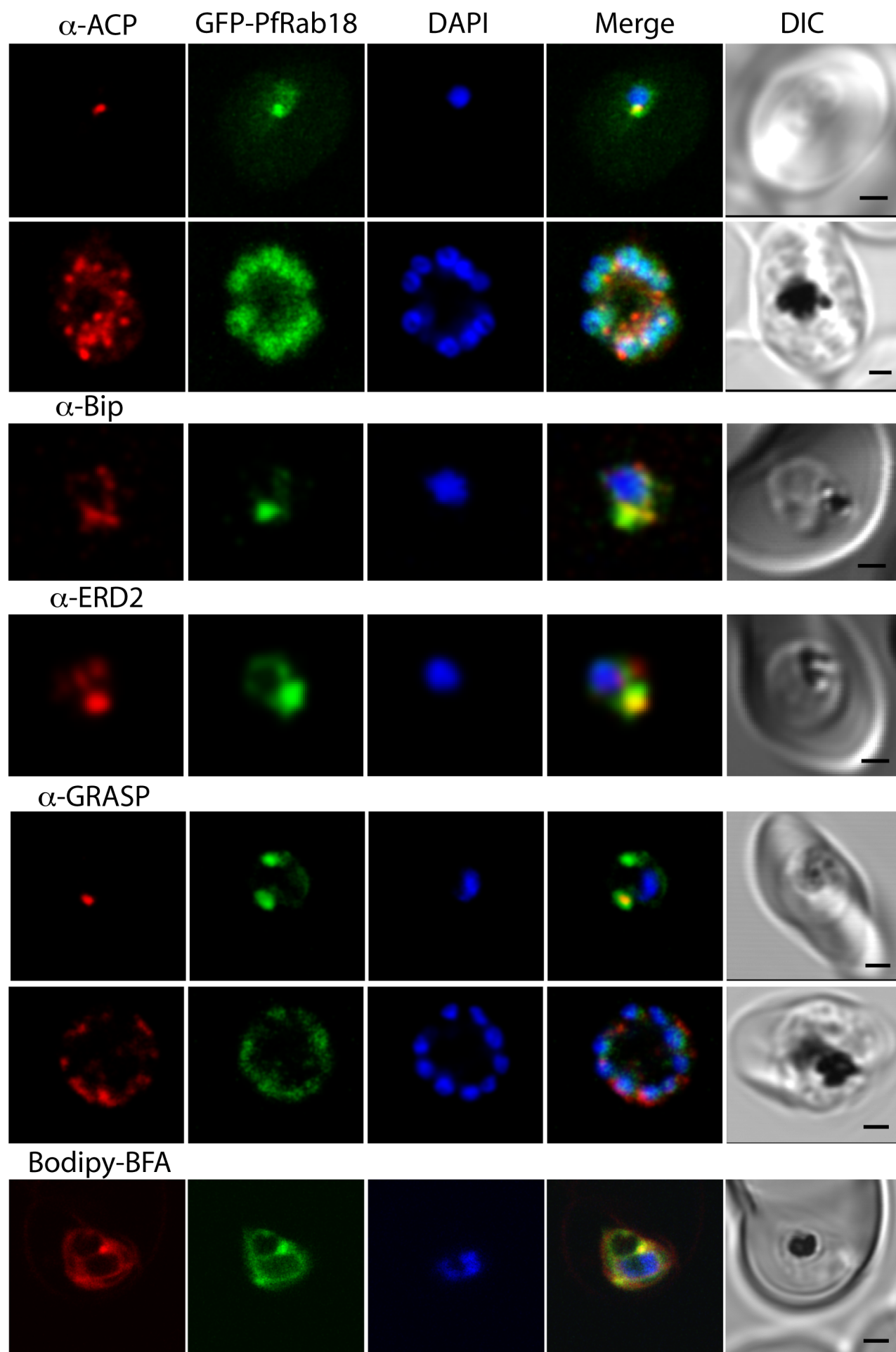

Supplement: S2 Fig — GFP-PfRab1A fluorescence is distinct from the localization of markers for the apicoplast (ACP), the ER (Bip), the Golgi (ERD2 and GRASP), as well as from staining of the ER/Golgi with Bodipy BFA. GFP-PfRab1A fluorescence is also distinct from the localization of markers for dense granules (RESA) or micronemes (AMA1 and EBA175). (PDF) [file pone.0158174.s002.pdf]
