# Supplementary material for: Plasmodium falciparum Rab1A Localizes to Rhoptries in Schizonts
Source: PLoS One. 2016 Jun 27;11(6):e0158174. doi: 10.1371/journal.pone.0158174 (PMC4922565; doi:10.1371/journal.pone.0158174)
Supplement: S1 Table — (PDF) [file pone.0158174.s003.pdf]

Protein name	Accessions	Peptide sequence	Mascot Ion score
heat shock protein hsp70 homologue	gi|11127605	SQIFSTAADNQTQVSIK	94.6
heat shock protein hsp70 homologue	gi|11127605	IINEPTAAALAFGLEK	80.3
heat shock protein hsp70 homologue	gi|11127605	NLTDSKNEAETLIYSSEK	76.1
Circumsporozoite protein-related antigen	gi|117393	KGSGEPLIDVHDLISDMIK	95.5
Circumsporozoite protein-related antigen	gi|117393	GSGEPLIDVHDLISDMIKK	48.8
Circumsporozoite protein-related antigen	gi|117393	GSGEPLIDVHDLISDMIK	46.4
multidrug resistance protein	gi|124506379	INNEGSYIIEQGTHDSLMK	66.2
multidrug resistance protein	gi|124506379	LYDPTEGDIIVNDSHNLK	58.8
rhoptry protein, putative	gi|124506661	KQDVLYETDKPQTMDEASYEETVDEDAHHVNEK	124
rhoptry protein, putative	gi|124506661	QHSAHFLDAIAEKDILEEK	57.1
Heat shock protein	gi|124506906	LYGQPGGPSPQPSGDEDVDSDEL	112
Heat shock protein	gi|124506906	DAEDWLNNNSNADSEALK	78.8
Heat shock protein	gi|124506906	LEATLHPTQTVFDVKR	77.2
Heat shock protein	gi|124506906	LEATLHPTQTVFDVK	71.5
Heat shock protein	gi|124506906	NLSVVHSTQIEIEDIVEGHNFSETLTR	70.6
Heat shock protein	gi|124506906	ITPSYVSFVDGER	60.8
Heat shock protein	gi|124506906	AKFEELNDDLFR	53.5
Heat shock protein	gi|124506906	NAVVTVPAYFNDAQR	48.6
Heat shock protein	gi|124506906	LKDLEAVCQPIIVK	47
Heat shock protein	gi|124506906	SLLPYEIVNNQGKPNIK	43.3
Heat shock protein	gi|124506906	DKDTTFAPEQISAMVLEK	41.8
hypothetical protein PFI1445w	gi|124507135	NIVSDALTSEEIKR	79.7
hypothetical protein PFI1445w	gi|124507135	LFEQIVDQIK	44.9
vacuolar proton-translocating ATPase	gi|124512642	SYQSMVDTYGVPR	62
vacuolar proton-translocating ATPase	gi|124512642	FLEENINKLPNVK	43.2
hypothetical protein PF13_0192	gi|124513440	MDLALNPTKL	46.5
hypothetical protein PF13_0192	gi|124513440	TNIYDSDEESESSEETSKDPYSSGPYTVDHK	42.3
elongation factor 1 alpha	gi|124513850	SVEMHKEVLEEARPGDNIGFNVK	59.8
elongation factor 1 alpha	gi|124513850	EVLEEARPGDNIGFNVK	39.9
elongation factor 1 alpha	gi|124513850	AGMVLNFAPSAVVSECK	35.5
elongation factor 1 alpha	gi|124513850	FTAQVIILNHPGEIK	34.4
hsp60	gi|124802320	QIAENAGHEGSVVAGNILK	69.6
hsp60	gi|124802320	YVDMIESGIIDPTK	33.9
histone H4	gi|124803496	TVTAMDIVYSLK	82.4
histone H4	gi|124803496	TVTAMDIVYSLKR	43.6
histone H4	gi|124803496	VFLENVIKDSIMYTEHAK	42.7
histone H2B	gi|124803500	SMNIMNSFLVDTFEK	56.5
histone H2B	gi|124803500	SRYDSYGLYIFK	34.4
ER-resident calcium binding protein	gi|124803623	LNDDQVKDILGLK	84.7
ER-resident calcium binding protein	gi|124803623	VYFDPAHESGAINVNEIK	78.3
ER-resident calcium binding protein	gi|124803623	VYFDPAHESGAINVNEIKENIFEGK	36.5
ER-resident calcium binding protein	gi|124803623	IAVTSLTDYGDVIR	32
endoplasmin homolog precursor	gi|124806075	GVVDSDDLPLNVSR	82.9
endoplasmin homolog precursor	gi|124806075	NNDDEQYIWESTADAK	79
endoplasmin homolog precursor	gi|124806075	DRDTLEEIEEGEKPTESMESHQYQTEVTR	68.8
endoplasmin homolog precursor	gi|124806075	WTLMNEQRPIWLR	61.8
endoplasmin homolog precursor	gi|124806075	LMDIIVNSLYTQK	60.6
endoplasmin homolog precursor	gi|124806075	NILSITDTGIGMTK	46.1
endoplasmin homolog precursor	gi|124806075	IDDLDPSIFETK	45.7
endoplasmin homolog precursor	gi|124806075	VDLINNLGTIAK	39.6
endoplasmin homolog precursor	gi|124806075	ALIDVISDTLK	39.5
vesicle-associated membrane protein	gi|124809346	NIHNMGYVDNNNINQDDPNLADGLK	38.3
vesicle-associated membrane protein	gi|124809346	YHELLNYCVFVDK	35.8
sortilin	gi|124809739	TVDKKPCECTPEDYECETGFTR	51
sortilin	gi|124809739	SAIEEDIAPFETNTEKR	31.6

hypothetical protein PF14_0593	gi|124810121	EGNNTSVNHDENSDADSLFNDHDENMQLK	70.9
hypothetical protein PF14_0593	gi|124810121	IDKPIAIVSVLGDMHTGK	52.7
Merozoite surface protein 1	gi|127331	TLSEVSIQTEDNYANLEK	105
Merozoite surface protein 1	gi|127331	TKVPNPLTISTTEMEK	75.1
Merozoite surface protein 1	gi|127331	VTTVVTPPQPDVTPSPLSVR	49.8
Merozoite surface protein 1	gi|127331	LLSTGLVQNFPNTIISK	48.7
Merozoite surface protein 1	gi|127331	ELKYPELFDLTNHMLTLSK	45.2
Merozoite surface protein 1	gi|127331	IDDYLINLK	38.2
Pr86 rhoptry precursor protein	gi|160328	NEGDLVAQKEEFEYDENMEK	86.3
Pr86 rhoptry precursor protein	gi|160328	SASVAGIVGADEEAPPAPK	64.2
Pr86 rhoptry precursor protein	gi|160328	NHIYELSHVNFCLLNPK	41.3
Pr86 rhoptry precursor protein	gi|160328	SASAAAILEEDDSKDDMEFK	39
Pr86 rhoptry precursor protein	gi|160328	ESMISTTFEQQKECLK	38.1
Pr86 rhoptry precursor protein	gi|160328	FHPNIDYLTLADGYK	31.7
